# Prospective GERiatric Observational (ProGERO) study: cohort design and preliminary results

**DOI:** 10.1186/s12877-020-01820-4

**Published:** 2020-10-27

**Authors:** Marcos Daniel Saraiva, Luís Fernando Rangel, Julia Lusis Lassance Cunha, Thereza Cristina Ariza Rotta, Christian Douradinho, Eugênia Jatene Bou Khazaal, Márlon Juliano Romero Aliberti, Thiago Junqueira Avelino-Silva, Daniel Apolinario, Claudia Kimie Suemoto, Wilson Jacob-Filho

**Affiliations:** Laboratorio de Investigacao Medica em Envelhecimento (LIM-66), Servico de Geriatria, Faculdade de Medicina, Hospital das Clinicas HCFMUSP, Universidade de Sao Paulo, Av. Dr. Eneas de Carvalho Aguiar 155, 8° andar, Setor Azul (Clinica Medica), LIM-66, Cerqueira Cesar, Sao Paulo, SP 05403-000 Brazil

**Keywords:** Cohort study, Outpatient, Older adult, Comprehensive geriatric assessment, Frailty, Disability, Survival

## Abstract

**Background:**

The demographic changes in Brazil as a result of population aging is one of the fastest in the world. The far-reaching new challenges that come with a large older population are particularly disquieting in low- and middle-income countries (LMICs). Longitudinal studies must be completed in LMICs to investigate the social and biological determinants of aging and the consequences of such demographic changes in their context. Therefore, we designed the Prospective GERiatric Observational (ProGERO) study, a longitudinal study of outpatient older adults in São Paulo, Brazil, to collect data both on aging and chronic diseases, and investigate characteristics associated with adverse outcomes in this population.

**Methods:**

The ProGERO study takes place in a geriatric outpatient clinic in the largest academic medical center in Latin America. We performed baseline health examinations in 2017 and will complete subsequent in-person visits every 3 years when new participants will also be recruited. We will use periodic telephone interviews to collect information on the outcomes of interest between in-person visits. The baseline evaluation included data on demographics, medical history, physical examination, and comprehensive geriatric assessment (CGA; including multimorbidity, medications, social support, functional status, cognition, depressive symptoms, nutritional status, pain assessment, frailty, gait speed, handgrip strength, and chair-stands test). We used a previously validated CGA-based model to rank participants according to mortality risk (low, medium, high). Our selected outcomes were falls, disability, health services utilization (emergency room visits and hospital admissions), institutionalization, and death. We will follow participants for at least 10 years.

**Results:**

We included 1336 participants with a mean age of 82 ± 8 years old. Overall, 70% were women, 31% were frail, and 43% had a Charlson comorbidity index score ≥ 3. According to our CGA-based model, the incidence of death in 1 year varied significantly across categories (low-risk = 0.6%; medium-risk = 7.4%; high-risk = 17.5%; *P* < 0.001).

**Conclusion:**

The ProGERO study will provide detailed clinical data and explore the late-life trajectories of outpatient older patients during a follow-up period of at least 10 years. Moreover, the study will substantially contribute to new information on the predictors of aging, senescence, and senility, particularly in frail and pre-frail outpatients from an LMIC city.

## Background

Brazil is currently facing one of the fastest population aging processes in the world [1]. It is expected that the percentage of older adults (over 60 years) in Brazil will rase from 13 to 36% from 2015 to 2050, while in the world, from 14 to 26% [1]. In France, for example, it took 100 years for the population aged 65 and over to double in size [2]. In Brazil, the same change will occur in 20 years, requiring rapid adaptation to this new reality [2]. However, contrary to what happened in more developed countries, the Brazilian demographic transition occurs in a context of unfavorable economic, social, and health conditions [1, 3, 4]. Although the poverty rate had decreased from 68% in 1970 to 31% in 2008, urbanization increased from 56 to 80%, and a public and universal health care system (Unified Health System) has been created, Brazil still has one of the world’s highest levels of socioeconomic inequality that are also seen across a range of health conditions and in access to and use of healthcare [3–5]. In Sao Paulo, the largest city in the country, inhabited by more than 12 million people, the aging index [(number of older adults aged 60 or older / number of people under the age of 15 years) × 100] has increased from 57% in 2010 to 80% in 2019 [6]. During the same period, the proportion of older adults (aged 60 years or more) living in the city increased from 12 to 15% [6].

Such a fast-paced demographic shift determines multifaceted changes that are not fully understood. The far-reaching challenges that come with a large older population are particularly disquieting in low- and middle-income countries (LMICs), where health systems are often ill-prepared to cope with the increasing burden of chronic diseases and high disability-adjusted life expectancy [3, 7]. Therefore, longitudinal studies are still necessary to investigate the social and biological determinants of adverse outcomes associated with aging, specifically predictors of disability and mortality, and its consequences in LMICs [3].

We have designed the Prospective GERiatric Observational (ProGERO) study, a longitudinal study of outpatient older adults of São Paulo, Brazil, to collect data both on aging and prevalent chronic diseases, and to investigate characteristics associated with adverse outcomes in this population. Our aim in this report was to describe our study design and share the baseline characteristics of our participants.

## Methods

### Study design and participants

The ProGERO is a prospective cohort study of older adults from an outpatient clinic at the Hospital das Clinicas of the University of Sao Paulo Medical School (HCFMUSP), in Sao Paulo, Brazil. This prospective cohort study aims to explore sociodemographic and clinical characteristics associated with adverse outcomes during the study follow-up, including falls, disability, emergency room (ER) visits, hospital admissions, institutionalization, and death, during a follow-up period of at least 10 years.

The HCFMUSP is the largest academic medical center in Latin America. This complex comprises eight specialty institutes that provide health care free of charge for 1.5 million persons (28% of them are older adults) every year, under the Brazilian Unified Health System. Physicians working at primary and secondary levels of care from all regions of the Sao Paulo metropolitan area can refer patients who are 60 years and older to the geriatric outpatient clinic. Older adults presenting geriatric conditions such as falls, multimorbidity, polypharmacy, cognitive impairment, functional disability, are considered a priority for reference to this specialized setting. The clinic operates 12 h a day, 5 days a week, offering comprehensive care guided by a multidisciplinary team composed of geriatricians, registered nurses, social workers, and psychologists. Medical appointments usually occur every 3 months.

In our recruitment, we invited every patient aged 60 years and over who had a medical appointment at the clinic between April and December 2017. We excluded subjects according to the following criteria: (1) need for immediate hospital admission or emergency care on baseline (e.g., hemodynamic instability, acute respiratory symptoms, delirium); (2) inability to be reached by telephone for follow-up assessments between visits; or (3) refusal to consent with the study.

**Fig. 1:**
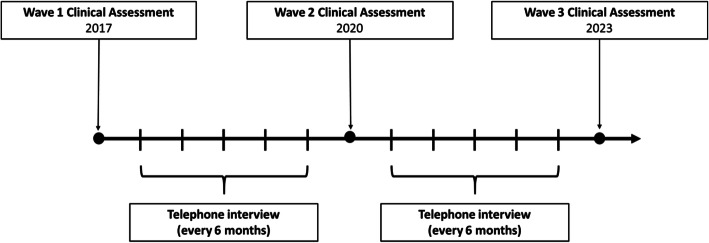
Schematic of study design. Clinical assessments are in-person with follow-up visits every 3 years for the reassessments of baseline characteristics and the inclusion of new participants

Eligible patients who consented to participate underwent a baseline clinical assessment with a standardized interview and physical examination. After recruitment, baseline characteristics will be reassessed every 3 years during in-person follow-up visits. We plan to invite new patients to participate in the study in each new wave of in-person visits and estimate to include approximately 700 new participants per recruitment cycle. We will also complete 6-month telephone interviews to collect data on our outcomes of interest between visits. Finally, we plan to follow participants for at least 10 years, or until their deaths. The initial study design, detailed in Fig. 1, will comprehend 3 waves of clinical assessment (2017, 2020, 2023), and we will follow participants until 2033.

### Clinical assessment

A trained multidisciplinary team of four registered nurses and four geriatricians completed the baseline clinical assessments. The nurses were responsible for undertaking the interviews and questionnaires. At the same time, geriatricians monitored data quality (reviewing missing data, elucidating queries during assessments), and reviewed electronic health records to collect data on multimorbidity and medications. The questionnaire protocol is described in detail in Additional file. When participants were unable to provide accurate responses due to overt dementia, we interviewed family members and caregivers with the closest contact to the participant to obtain the best information available.

We managed our database using Research Data Capture (REDCap) resources [8]. All identifiable information was kept in secure data servers, and access was restricted to our research team, safeguarding confidentiality and anonymity.

#### Demographics

We collected the following sociodemographic data: age; sex; race/ethnicity; marital status; level of literacy; occupation; annual household income per capita, expressed both as a continuous variable and as categories according to the Brazilian minimum wage in 2017 [1 minimal wage = 4000 United States dollar (USD) per year]; and neighborhood. We also recorded whether participants lived alone or with other persons.

#### Multimorbidity and medications

We measured multimorbidity using the Charlson comorbidity index [9] and the Functional Comorbidity Index (FCI) [10], based on information retrieved from medical records. We also used medical records to compile the lists of medications in use.

The Charlson comorbidity index includes 19 clinical conditions, with various scoring weights, and the final score is defined by the total sum of items (range: 0–37 points; 37 = worst) [9]. We analyzed the Charlson comorbidity index both as a continuous variable and stratified in ordinal categories (0, 1–2, and ≥ 3 points) [11]. The FCI is a comorbidity scale designed to predict functional decline. It includes 18 clinical conditions, and its score corresponds to the total disease count (range: 0–18; 18 = worst) [10].

#### Anthropometry, physical examination, and sensory evaluation

Anthropometric and physical examination measures included: blood pressure, pulse rate, weight, height, and calf circumference.

Blood pressure and pulse rate were measured at the heart level, using an electronic manometer and a standardized cuff (Omron Hem-7113 Automatic Blood Pressure Monitor, Omron Healthcare Co., Ltd.). Values were recorded after 5 min of rest while sitting. Three readings were taken in succession, with at least one-minute intervals, and the average was used for the analyses [12].

We asked participants to wear light clothing and no shoes when anthropometric measures were taken. We calculated body mass index (BMI) using the metric system (kg/m^2^) and measured the calf circumference (cm) using an inelastic tape placed on the broadest possible section of the left calf [13].

Finally, we screened for the presence of visual and auditive deficits (“yes” or “no”) using the following questions, extracted from the Alzheimer’s Disease Cooperative Study - Activities of Daily Living - Prevention Instrument (ADCS-ADL-PI) Questionnaire [14]: (1) “Can you see well enough to recognize a friend across the street?”; and (2) “Can you usually hear and understand another person when they talk in a normal voice?”

#### Comprehensive geriatric assessment

The 10-min Targeted Geriatric Assessment (10-TaGA) [13] is a validated multi-domain hands-on instrument that was developed to screen geriatric syndromes and estimate the global impairment of patients, using the cumulative deficit model. In previous research, 10-TaGA provided adequate validity and good accuracy in discriminating between frail and non-frail individuals and good predictive power for one-year mortality, disability and hospitalization [13, 15, 16]. This quick and easy-to-administer CGA-based tool assembles objective measures and self-reported information in an efficient method that evaluates 10 health domains: (1) social support (living arrangements and availability of help) [17]; (2) emergency department visits and hospitalizations in the previous 6 months; (3) the number of falls in previous 12 months; (4) the number of medications; (5) dependence in activities of daily living (ADLs) (Katz index) [18]; (6) 10-point Cognitive Screener (10-CS) [19]; (7) self-rated health; (8) 4-item Geriatric Depression Scale (GDS-4) score [20]; (9) nutritional status (weight loss in the previous 12 months, BMI and calf circumference); (10) gait speed [13]. Each domain is categorized and scored as normal (0 points), mild impairment (0.5 points), or severe impairment (1 point), based on validated cut-off points against conventional and more extended instruments that are widely used in practice to assess each geriatric condition [13]. While 10-TaGA captures health deficits in multiple domains using rapid geriatric measures, which might be considered superficial, the tool proved to be a practical and efficient method to introduce the CGA in busy clinical settings where time-consuming instruments are unfeasible [13, 15, 16]. Based on previous work demonstrating the good predictive power of 10-TaGA for one-year mortality, we classified participants as having low (0–0.24), medium (0.25–0.49), or high (0.50–1) risk of death [16].

#### Functional status

We examined detailed information on functional disability using the Brazilian version of the Older Americans Resources and Services Multidimensional Functional Assessment Questionnaire (BOMFAQ) [21] and the Katz index [18]. The BOMFAQ (range: 0–30; 30 = worst) evaluates dependency in 15 ADLs and instrumental activities of daily living (IADLs). For each item, a score from zero to two is assigned (0: unable to perform the activity; 1: needs supervision or help to perform the activity; 2: completely independent to perform the activity) [21].

We additionally used the Katz index to evaluate ADLs (feeding, dressing, bathing, toileting, transferring, and continence). Each activity is scored as either zero (unable to perform the activity) or one (completely independent to perform the activity) [13, 18].

#### Pain

We screened for pain complaints and their intensity using the 5-point Verbal Descriptor Scale: no pain; mild pain; moderate pain; severe pain; or worst possible pain [22]. Participants identified as having any pain were also asked the following question: “Did the pain occur on most days in the past three months?”. A negative answer defined sporadic pain, while a positive answer defined persistent pain [23, 24]. We further investigated participants with persistent pain using the multidimensional Geriatric Pain Measure [25, 26].

#### Frailty

We defined frailty using the Study of Osteoporotic Fractures (SOF) [27] index for frailty, and the FRAIL scale [28, 29]. The SOF index includes three items: weight loss of 5% or more; inability to rise from a chair five times; and reduced energy level. The score ranges from zero to three points, and classifies patients as: robust (0 points); pre-frail (1 point); or frail (2–3 points) [27]. The FRAIL scale includes five mnemonic questions on fatigue, resistance, ambulation, illnesses, and loss of weight. The score ranges from zero to five points, and classifies patients as: robust (0 points); pre-frail (1–2 points); or frail (≥3 points) [28, 29]. Detailed instructions to administer the SOF and FRAIL indexes can be found in the assessment form in the Additional File. Although the phenotypic criteria for the diagnosis of frailty [30] were not used in our study, recent studies have shown that the SOF index and FRAIL scale have similar performances to predict adverse outcomes in vulnerable older adults [31–33]. Moreover, both are quick and easy-to-administer frailty screening indexes and feasible in the scenario of a busy clinical setting, particularly in the context of an LMIC city.

#### Physical performance

We measured gait speed instructing participants to walk 4.5 m at their usual pace and used the faster of two measurements in our analyses. Participants were allowed the use of assistive devices whenever necessary [13].

We measured handgrip strength using a Saehan dynamometer. We requested that participants sit on armless chairs, with their spines erect, shoulders positioned in adduction and neutral rotation, elbows flexed at 90°, forearms in half pronation, and neutral wrists. They would then squeeze the device using their dominant hand, applying their maximum strength. We used the mean value of three measurements in our analyses [34].

Finally, we requested that those who were able to sit and stand independently do the chair-stands test. We asked that participants do their best to complete five sit-to-stand repetitions, without the help of the arms, and recorded the total time in seconds [35].

### Telephone follow-up

Investigators blinded to the clinical assessment conduct telephone interviews every 6 months. They interview participants (or their proxy) using structured questionnaires designed to collect data on falls, functional status, frailty, pain, use of health services, institutionalization, and death (including date of death). In cases of death, we checked the information about the date, place, and leading cause of death with a family member. We also have the possibility to verify more information about the circumstances of death using the hospital electronic health records and the Registry of Death of Sao Paulo city, which can be assessed for the research purpose upon request. The telephone interview takes from 5 to 10 min. In case we could not reach a patient by phone, we use the following strategies (1) contact another family member or caregiver; (2) send emails and text messages asking for information; (3) proceed an in-person interview during his/her medical appointment in the geriatric clinic; (4) search for news and events in medical records. We succeeded in contacting more than 99% of participants using this approach during the first-year follow-up.

Medical investigators adjudicate the quality of the telephone interviews. The adjudication process includes the review of missing data, information reliability and periodic feedback meetings between medical investigators and research assistants.

### Outcomes

Our primary longitudinal outcomes are the time to death (survival) and time to new ADL disability. We defined new ADL disability for patients having developed the need for assistance in a previously preserved ADL (including eating, transferring, dressing, toileting, and bathing), as compared to baseline. Those participants who need help in all ADLs at baseline (“complete ADL disability”) are excluded from the incident disability analysis. Also, we will not assess incontinence as a measure of disability, given the high frequency and multifaceted meaning of this condition in older adults [36].

We additionally selected the following secondary outcomes: hospital admissions, defined as planned or unplanned hospital stays for 24 h or more; ER visits, defined as any ER visit during the follow-up; falls, defined as an unintentional displacement of the body to a lower level, with an inability to correct said displacement on time [37]; and institutionalization. We will also measure BOMFAQ scores in our telephone follow-up and use it as a repeated measures outcome.

### Analyses plan

#### Sample size calculations

We calculated our sample size based on recent work that determined the patients’ mortality risk according to different levels of 10-TaGA score [16]. We used the log-rank test to compare the 12-month survival among three groups (low, medium, and high risk), with a distribution of 1:2:1, respectively. Assuming a one-year mortality rate of 2.5% in the lowest risk group [16], an alternative bilateral hypothesis, an alpha error of 0.05%, a beta error of 0.20 and an estimated sample loss of 15%, we projected that a total sample of 1081 participants would be required to detect 5% differences between the groups.

#### Statistical analyses

We described continuous variables using means and standard deviations (SD) or medians and interquartile ranges (IQR), according to their distribution. We reported categorical variables using counts and percentages. Further, we presented the characteristics of participants and one-year incidence of death across the three 10-TaGA categories. We used a one-way analysis of variance (ANOVA) or its non-parametric equivalent (Kruskal-Wallis) to compare continuous variables across categories. We used the trend chi-square test (χ2) to compare independent proportions. For missing data, we used the complete-case analysis approach, as only two participants didn’t complete the first-year telephone follow-up. Although we did not consider variables such as the recruitment wave and significant changes in the care provided at the geriatric outpatient clinic, future analyses encompassing the different study waves may include those factors as possible confounders. All statistical tests were two-tailed, and an alpha level of 0.05 was used to determine significance.

### Ethics

All subjects provided written informed consent to participate in the study and also given permission to link their clinical information from the hospital enterprise electronic health record reporting database (Prontmed®) with the research data. In those participants with overt dementia, defined by a medical diagnosis or 10-CS score equal to zero, the consent was obtained from a proxy (*N* = 532). The study and informed consent form have been approved by the local ethics committee of São Paulo University School of Medicine (CAAE: 65809517.3.0000.0068).

Attending physicians working in our clinic do not have access to the study data and were not involved in participant recruitment or data collection. The decision to participate did not affect the patients’ standard of care at the geriatric outpatient clinic. Participants were also advised that they could leave the study at any point.

### Data sharing

To enhance reporting transparency, this study will be reported in accordance with the Strengthening the Reporting of Observational Studies in Epidemiology Statement (STROBE): Guidelines for Reporting Observational Studies [38]. Data and resources will be shared with other eligible investigators through academically established means. The datasets used during the study will be available from the corresponding author on reasonable request.

## Results

**Fig. 2:**
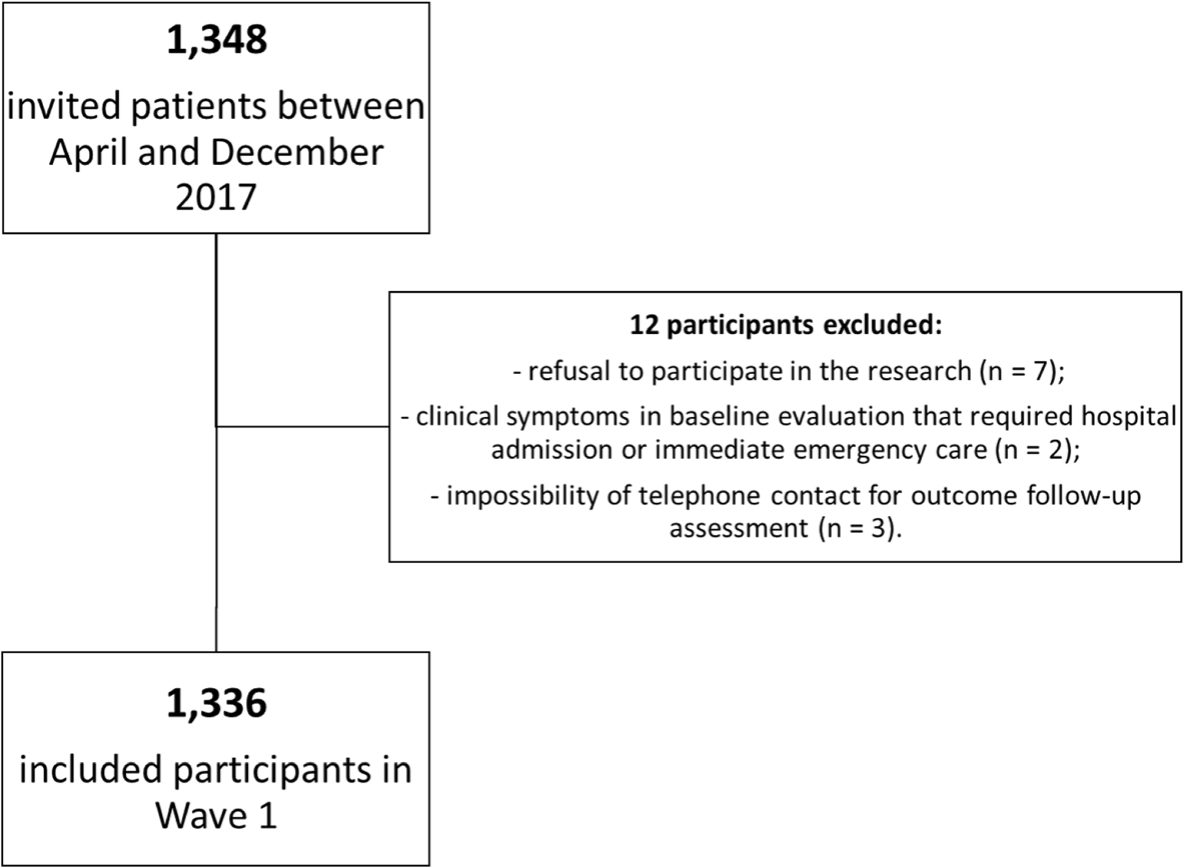
Flowchart of Wave 1 Baseline Clinical Assessment of Prospective GERiatric Observational (ProGERO) Study baseline participants (2017)

In April 2017, we had 1468 older patients registered in the geriatric outpatient clinic. We identified 1348 study candidates in our outpatient clinic between April and December 2017 and 108 participants were unable to be reached during the study period. We did not include 12 participants for the following reasons: refusal to participate in the study (*n* = 7), baseline clinical symptoms requiring hospital admission or immediate emergency care (*n* = 2), or inability to comply with telephone follow-up interviews (*n* = 3). Consequently, we included and assessed 1336 older adults on baseline (Fig. 2).

**Fig. 3:**
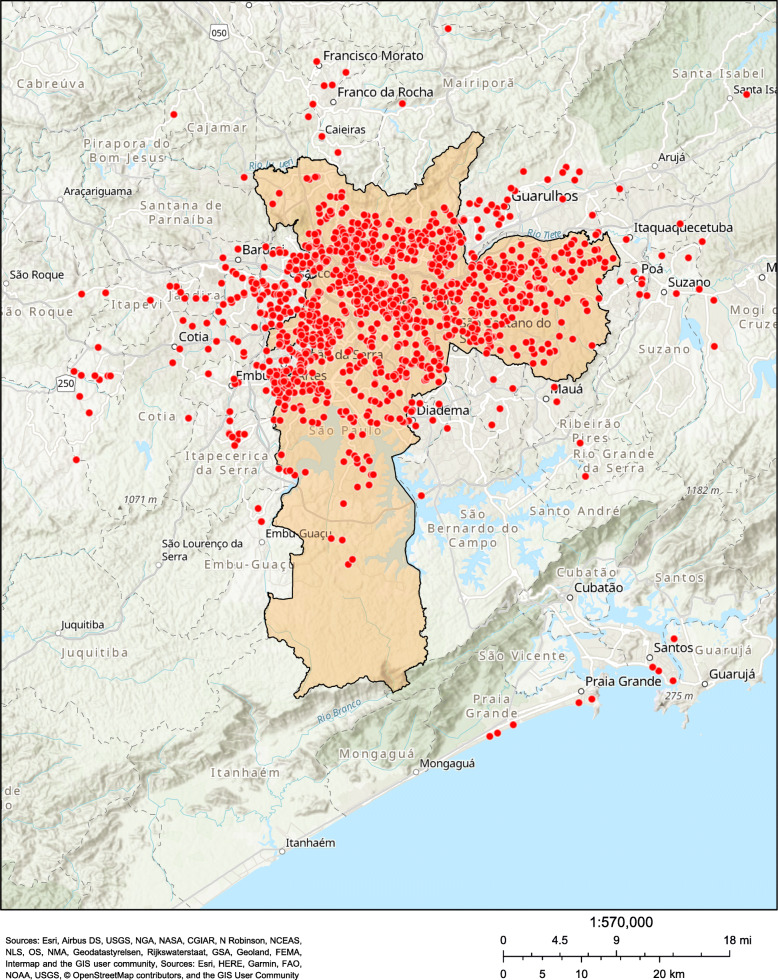
Distribution of Prospective GERiatric Observational (ProGERO) Study baseline participants (2017) in the metropolitan area of São Paulo, Brazil. Sources: Esri, Airbus DS, USGS, NGA, NASA, CGIAR, N Robinson, NCEAS, NLS, OS, NMA, Geodatastyrelsen, Rijkswaterstaat, GSA, Geoland, FEMA, Intermap and the GIS user community, Sources: Esri, HERE, Garmin, FAO, NOAA, USGS, © OpenStreetMap contributors, and the GIS User Community

Participants had a mean (SD) age of 82 ± 8 years, 70% were women, 57% identified as being white, 52% were widowed, and 52% had an annual household income per capita between 4000 and 8000 USD (Table 1). Overall, 83% of our participants lived in Sao Paulo city, coming from all the different regions of the city. Figure 3 illustrates an even broader area represented in the ProGERO Study, with patients living in at least 28 cities in the metropolitan area of Sao Paulo.

**Table 1:** Baseline sample sociodemographic characteristics (2017) of Prospective GERiatric Observational (ProGERO) Study, according to the 10-min Target Geriatric Assessment (10-TaGA) risk categories (*n* = 1336)

Sociodemographic characteristics	10-TaGA risk categories				
	Total	Low (*n* = 160)	Medium (*n* = 735)	High (*n* = 441)	***P***-value
**Age (years), mean (SD)**	82.22 (7.58)	78.74 (7.67)	82.35 (7.34)	83.26 (7.59)	**< 0.001**
**Female, n (%)**	938 (70.21)	97 (60.62)	520 (70.75)	321 (72.79)	**0.014**
**Ethnicity, n (%)**					
**White**	758 (56.74)	100 (62.50)	415 (56.46)	243 (55.10)	0.488
**Black**	376 (28.14)	17 (10.62)	85 (11.56)	51 (11.57)	
**Mixed**	153 (11.45)	35 (21.88)	207 (28.17)	134 (30.39)	
**Asian**	45 (3.37)	8 (5.00)	26 (3.54)	11 (2.49)	
**Indigenous**	4 (0.30)	0 (0.00)	2 (0.27)	2 (0.45)	
**Marital status, n (%)**					
**Widowed**	698 (52.25)	57 (35.62)	391 (53.20)	250 (56.69)	**< 0.001**
**Married**	457 (34.21)	83 (51.87)	249 (33.88)	125 (28.34)	
**Single**	92 (6.89)	11 (6.88)	42 (6.12)	36 (8.16)	
**Divorced**	89 (6.66)	9 (5.53)	50 (6.80)	30 (6.81)	
**Level of literacy (years), median (IQR)**	4 (1–5)	4 (3–10)	4 (2–5)	4 (1–4)	**< 0.001**
**Annual household income per capita**^**a**^**, n (%) (*N*** **= 1318)**					
**< 4000 USD**	345 (26.18)	38 (24.36)	191 (26.27)	116 (26.67)	**0.028**
**4000–8000 USD**	684 (51.90)	75 (48.08)	364 (50.07)	245 (56.32)	
**> 8000 USD**	289 (21.92)	43 (27.56)	172 (23.66)	74 (17.01)	

^a^annual household income was classified according to the Brazilian minimum wage in 2017 (1 minimal wage = 4000 USD per year)

*10-TaGA* 10-min Target Geriatric Assessment, *SD* standard deviation, *IQR* interquartile range, *USD* United States dollar

To compare the 10-TaGA risk categories, we used one-way analysis of variance (ANOVA), its non-parametric equivalent (Kruskal-Wallis), and the trend chi-square test. All statistical tests were two-tailed, and an alpha level of 0.05 was used to determine significance

The 10-TaGA scores ranged from 0 to 0.9, with a mean of 0.4 ± 0.2 points. According to our risk categories, 160 (12%) participants were classified as having low risk (0–0.24), 735 (55%) had medium risk (0.25–0.49), and 441 (33%) had high risk (0.5–1.0) of death.

Participants had a mean BMI of 27.2 ± 5.2 kg/m^2^, walking speed of 0.7 ± 0.2 m/s, and handgrip of 13.5 ± 7.5 kg. We observed that 59% of the participants had difficulty in at least one ADL on the baseline, 31% were frail according to the SOF index criteria, 21% were frail by the FRAIL scale criteria, and 43% had Charlson comorbidity index scores > 2 points. The most prevalent comorbidities were hypertension (81%), persistent pain (44%), diabetes (36%), dementia (37%), congestive heart failure (21%), chronic kidney disease (21%), cerebrovascular disease (20%), coronary artery disease (20%), cancer (excluding non-melanoma skin cancer) (14%), and chronic obstructive pulmonary disease (7%). Visual impairment was verified in 66% of our sample, and hearing impairment in 78%. Also, 15% reported being current or past smokers. Additional characteristics of our population can be found in Table 2. Also, Table 3 summarizes the baseline characteristics of participants in the ProGERO study and other LMICs cohort studies.

**Table 2:** Baseline clinical and functional sample characteristics (2017) of Prospective GERiatric Observational (ProGERO) Study, according to the 10-min Target Geriatric Assessment (10-TaGA) risk categories (*n* = 1336)

Clinical and functional characteristics	10-TaGA risk categories				
	Total	Low (*n* = 160)	Medium (*n* = 735)	High (*n* = 441)	***P*** value
**BOMFAQ, median (IQR)**	21 (15–26)	28 (26–30)	22 (18–26)	14 (9–19)	**< 0.001**
**Katz, median (IQR)**	5 (4–6)	6 (6–6)	5 (5–6)	3 (1–5)	**< 0.001**
**SOF index, n (%)**					
**Robust**	442 (33.08)	115 (71.88)	278 (37.82)	49 (11.11)	**< 0.001**
**Pre-frail**	474 (35.48)	42 (26.25)	298 (40.55)	134 (30.39)	
**Frail**	420 (31.44)	3 (1.87)	159 (21.63)	258 (58.50)	
**FRAIL scale, n (%)**					
**Robust**	305 (22.83)	66 (41.25)	167 (22.72)	72 (16.33)	**< 0.001**
**Pre-frail**	709 (53.07)	85 (53.13)	421 (57.28)	203 (46.03)	
**Frail**	322 (24.10)	9 (5.62)	147 (20.00)	166 (37.64)	
**Handgrip (kg), mean (SD) (*n*** **= 1136)**	13.48 (7.53)	18.23 (7.69)	13.49 (7.53)	11.14 (6.25)	**< 0.001**
**Inability to do the 5-repetition Sit-to-stand test, n (%)**	572 (42.81)	13 (8.13)	243 (33.06)	316 (71.66)	**< 0.001**
**Walking speed (m/s), mean (SD) (*n*** **= 1012)**	0.69 (0.22)	0.90 (0.20)	0.68 (0.19)	0.56 (0.22)	**< 0.001**
**BMI (kg/m**^**2**^**), mean (SD) (*n*** **= 1199)**	27.15 (5.24)	27.56 (4.15)	27.44 (5.30)	26.38 (5.51)	**< 0.001**
**Calf circumference (cm), mean (SD) (*n*** **= 1325)**	33.70 (4.44)	34.95 (3.53)	34.28 (4.32)	32.28 (4.57)	**0.001**
**Charlson comorbidity index, n (%)**					
**0 points**	192 (14.37)	51 (31.87)	111 (15.10)	30 (6.80)	**< 0.001**
**1–2 points**	568 (42.51)	69 (43.13)	324 (44.08)	175 (39.68)	
**≥ 3 points**	575 (43.11)	40 (25.00)	300 (40.82)	236 (53.52)	
**FCI, median (IQR)**	3 (2–4)	2.5 (1–4)	3 (2–4)	3 (2–5)	**< 0.001**
**10-CS punctuation, n (%)**					
**Uncapable**	227 (17.00)	0 (0)	75 (10.20)	152 (34.47)	**< 0.001**
**≥8 (normal)**	437 (33.70)	113 (70.63)	267 (36.33)	57 (12.92)	
**6–7 (possible CI)**	280 (20.96)	28 (17.50)	180 (24.49)	72 (16.33)	
**0–5 (probable CI)**	392 (29.34)	19 (11.87)	213 (28.98)	160 (36.28)	

*10-TaGA* 10-min Target Geriatric Assessment, *BOMFAQ* Brazilian version of the Older Americans Resources and Services Multidimensional Functional Assessment Questionnaire, *IQR* interquartile range, *SOF* Study of Osteoporotic Fractures, *SD* standard deviation, *BMI* body mass index, *FCI* Functional Comorbidity Index, *10-CS* 10-point Cognitive Screener, *CI* cognitive impairment

To compare the 10-TaGA risk categories, we used one-way analysis of variance (ANOVA), its non-parametric equivalent (Kruskal-Wallis), and the trend chi-square test. All statistical tests were two-tailed, and an alpha level of 0.05 was used to determine significance

**Table 3:** Sociodemographic and clinical characteristics of low- and middle-income countries (LMICs) main cohort studies

Sociodemographic and clinical characteristics	LMICs cohort studies				
	ProGERO	ELSI-Brazil	SABE	MHAS	CHARLS
**Country**	Brazil	Brazil	Brazil	Mexico	China
**Total participants**	1336 (≥ 60 years)	9412 (≥ 50 years)	2143 (≥ 60 years)	15,186 (≥ 50 years)	17,708 (≥ 45 years)
**Year of Baseline Assessment**	2017	2015–2016	2000	2001	2011–2012
**Age (years)**	Mean (SD): 82.2 (7.58)	Mean (CI): 62.9 (62.1–63.8)	Mean (range): 68 (60–100)	Mean (SD): Men: 62.3 (9.59); Women: 62.3 (9.67)	Mean (SD): Men: 59.8 (0.24); Women: 59.6 (0.29)
**Female, %**	70.2	54.0	58.6	53.3	49.4
**Marital status, %**					
**Widowed**	52.3	14.7	62.9	Men: 10.8; Women: 27.5	20.6
**Married**	34.2	63.5	30.0	Men: 78.8; Women: 55.4	77.2
**Single/ Divorced**	13.5	21.8	7.1	Men: 10.4; Women: 17.1	2.2
**Hypertension, %**	80.8	63.2	53.3	Men: 26.4; Women: 44.1	32.1
**Diabetes, %**	35.7	15.8	17.6	Men: 12.5; Women: 16.7	7.2
**Cardiovascular disease, %**	19.6	11.7	19.5	Men: 2.6; Women: 2.9	16.3
**Cerebrovascular disease, %**	19.9	5.3	8.2	Men: 2.6; Women: 2.6	3.1
**Cancer, %**	14.0	5.3	4.6	Men: 1.2; Women: 2.5	0.9
**Difficulty in at least 1 ADL, %**	58.9	23.2	22.1	Men: 8.6; Women: 10.8	18.8
**Frailty, %**	31.4	9	8.5	24.9	7.0

*LMICs* low- and middle-income countries, *ProGERO* Prospective GERiatric Observational Study, *ELSI-Brazil* The Brazilian Longitudinal Study of Aging, *SABE* The health, well-being and aging project, *CHARLS* The China Health and Longitudinal Study, *MHAS* The Mexican Health and Aging Study, *SD* standard deviation, *CI* confidence interval, *ADL* activities of daily living

ELSI: The Brazilian Longitudinal Study of Aging is a nationally representative study of 9412 people aged 50 years or older, residing in 70 municipalities across the 5 Brazilian regions. ELSI-Brazil allows investigations of the aging process, its health, psychosocial and economic determinants, and societal consequences. The baseline examination was conducted in 2015–2016 [3, 40, 39, 41]

SABE: The health, well-being and aging project was coordinated by the Pan American Health Organization and aimed to collect information about the living conditions of the elderly population (aged 60 and older) in urban metropolitan areas in seven Latin American countries, and to investigate cohort, socioeconomic and gender differences in relation to health status, as well as use and access to health care. In Table 3, we described data from 2143 participants of the city of São Paulo [43, 44]

CHARLS: The China Health and Longitudinal Study is a nationally representative longitudinal survey of the middle-aged and elderly population (45+) in China along with their spouses, which includes an assessment of the social, economic, and health circumstances of community-residents. The national baseline survey of CHARLS was conducted between June 2011 and March 2012 on 17,708 respondents. In Table 3, we described data from 5301 adults aged 60 years old or more who had complete data on frailty components [47, 48]

MHAS: The Mexican Health and Aging Study was designed to prospectively evaluate the impact of diseases on the health, function, and mortality of adults over the age of 50 in both urban and rural areas of Mexico. The MHAS 2001 baseline is a nationally and urban-rural representative survey of 15,186 individuals born in 1951 or earlier [50, 51]

Finally, the one-year incidence of death was considerably different across the 10-TaGA categories (low-risk = 0.6%; medium-risk = 7.4%; high-risk = 17.5%; *P* < 0.001).

## Discussion

In this study, we describe the design and preliminary results of the ProGERO study, which aims to investigate the determinants of aging, senescence, and senility in outpatient older adults from an LMIC city.

In comparison with other cohort studies of older adults in São Paulo [42–45], Brazil [3, 39–41] and LMICs [46–51], our participants were older, had a more significant burden of disease, and a higher prevalence of frailty (Table 3). The result was expected since our sample is mostly comprised of multimorbid older adults, followed in an academic medical center. Despite the profile of ProGERO participants bringing some drawbacks to our study, we have some advantages over other LMICs population-based cohorts in a few aspects: (1) superior statistical power to explore patient-centered outcomes, considering the elevated incidence of these outcomes in a cohort of vulnerable older adults; (2) higher prevalence of the oldest old, ensuring a favorable opportunity to study these fast-growing age group; (3) higher accuracy and availability of clinical information provided by a teaching environment; and (4) small loss of outcome data guaranteed by telephone interviews between in-person visits. Furthermore, the ProGERO study is a prospective cohort study with an expected follow-up of several years. Therefore, our investigation may assist in determining reliable predictors of adverse outcomes in outpatient older adults, and in characterizing their functional trajectories over time.

Nevertheless, our results should be interpreted in the context of our limitations. We used convenience and non-random sample from a geriatric outpatient clinic. Despite including patients from several different areas of Sao Paulo, the sociodemographic and clinical profile of our sample might limit the external generalizability of our results. In comparison to the sociodemographic characteristics of older adults living in São Paulo, our sample has a higher proportion: of women (70% versus 59%); of older adults aged 75 years or more (84% versus 24,6%); of at least one ADL disability (58,9% versus 18,5%); of older adults with 4 or more years of schooling years (58% versus 42%); of black and mixed ethnicity (39,5% versus 24,7%); and of older adults with annual household income over 2000 USD (96,6% versus 88,4%) [8]. Moreover, the proportion of low-risk participants was lower than we estimated when calculating the sample size, mostly because of the clinical profile of our sample. Even though we acknowledge that the results built on our cohort need to take account of the particular characteristics of our sample, we also consider that we can offer crucial insights into the aging process of pre-frail and frail people in LMICs.

## Conclusions

In conclusion, the ProGERO study is a prospective cohort study that will collect and explore comprehensive, long-term clinical data of geriatric outpatients from an LMIC city. The watchful follow-up of this convenience sample will offer clinicians and researchers cutting-edge knowledge on how sociodemographic and clinical factors affect the functional trajectories of outpatient older adults with multimorbidity.

## Supplementary information

**Additional file 1.** Research Protocol – ProGERO Study.

## Data Availability

The datasets used and analyzed in the study are available from the corresponding author on reasonable request.

## Abbreviations

10-CS: 10-point Cognitive Screener
10-TaGA: 10-min Target Geriatric Assessment
ADCS-ADL-PI: Alzheimer’s Disease Cooperative Study - Activities of Daily Living - Prevention Instrument
ADL: Activities of daily living
BMI: Body mass index
BOMFAQ: Brazilian version of the Older Americans Resources and Services Multidimensional Functional Assessment Questionnaire
CGA: Comprehensive geriatric assessment
ER: Emergency room
FCI: Functional Comorbidity Index
GDS-4: 4-item Geriatric Depression Scale
HCFMUSP: Hospital das Clinicas, University of São Paulo Medical School
IADL: Instrumental activities of daily living
IQR: Interquartile range
LMICs: Low- and middle-income countries
ProGERO: Prospective GERiatric Observational
REDCap: Research Electronic Data Capture
SD: Standard deviation
SOF: Study of Osteoporotic Fractures
STROBE: Strengthening the Reporting of Observational Studies in Epidemiology Statement
USD: United States dollar

## References

[1] Desa U (2015). United nations department of economic and social affairs, population division. world population prospects: The 2015 revision, key findings and advance tables. Technical Report. Working Paper No. ESA/P/WP. 241.

[2] Global health and aging. WHO US National Institute of Aging. 2011;:273–7. https://www.who.int/ageing/publications/global_health/en/.

[3] Lima-Costa MF, de Andrade FB, de Souza Jr PRB, Neri AL, Duarte YA, Castro-Costa E (2018). The Brazilian longitudinal study of aging (ELSI-Brazil): objectives and design. Am J Epidemiol.

[4] Lima-Costa MF, Facchini LA, Matos DL, Macinko J (2012). Changes in ten years of social inequalities in health among elderly Brazilians (1998–2008). Rev Saude Publica.

[5] Paim J, Travassos C, Almeida C, Bahia L, Macinko J (2011). The Brazilian health system: history, advances, and challenges. Lancet (London, England).

[6] Indicadores Sociodemográficos da População Idosa na Cidade de São Paulo (2019). Secretaria Municipal de Direitos Humanos e Cidadania; Coordenadoria de Políticas para Pessoa Idoso - São Paulo.

[7] Salomon JA, Wang H, Freeman MK, Vos T, Flaxman AD, Lopez AD (2012). Healthy life expectancy for 187 countries, 1990–2010: a systematic analysis for the Global Burden Disease Study 2010. Lancet (London, England).

[8] Harris PA, Taylor R, Thielke R, Payne J, Gonzalez N, Conde JG (2009). Research electronic data capture (REDCap)--a metadata-driven methodology and workflow process for providing translational research informatics support. J Biomed Inform.

[9] Charlson ME, Pompei P, Ales KL, MacKenzie CR (1987). A new method of classifying prognostic comorbidity in longitudinal studies: development and validation. J Chronic Dis.

[10] Groll DL, To T, Bombardier C, Wright JG (2005). The development of a comorbidity index with physical function as the outcome. J Clin Epidemiol.

[11] Sundararajan V, Henderson T, Perry C, Muggivan A, Quan H, Ghali WA (2004). New ICD-10 version of the Charlson comorbidity index predicted in-hospital mortality. J Clin Epidemiol.

[12] Consortium T (2018). Recommended standards for assessing blood pressure in human research where blood pressure or hypertension is a major focus. Clin Exp Hypertens.

[13] Aliberti MJR, Apolinario D, Suemoto CK, Melo JA, Fortes-Filho SQ, Saraiva MD (2018). Targeted geriatric assessment for fast-paced healthcare settings: development, validity, and reliability. J Am Geriatr Soc.

[14] Galasko D, Bennett DA, Sano M, Marson D, Kaye J, Edland SD (2006). ADCS prevention instrument project: assessment of instrumental activities of daily living for community-dwelling elderly individuals in dementia prevention clinical trials. Alzheimer Dis Assoc Disord.

[15] Aliberti MJR, Covinsky KE, Apolinario D, Smith AK, Lee SJ, Fortes-Filho SQ (2019). 10-minute targeted geriatric assessment predicts disability and hospitalization in fast-paced acute care settings. J Gerontol A Biol Sci Med Sci.

[16] Aliberti MJR, Covinsky KE, Apolinario D, Lee SJ, Fortes-Filho SQ, Melo JA (2019). A 10-min targeted geriatric assessment predicts mortality in fast-paced acute care settings: A prospective cohort study. J Nutr Health Aging.

[17] Sherbourne CD, Stewart AL (1991). The MOS social support survey. Soc Sci Med.

[18] Katz S, Akpom CA (1976). A measure of primary sociobiological functions. Int J Health Serv.

[19] Apolinario D, Lichtenthaler DG, Magaldi RM, Soares AT, Busse AL, das Gracas Amaral JR (2016). Using temporal orientation, category fluency, and word recall for detecting cognitive impairment: the 10-point cognitive screener (10-CS). Int J Geriatr Psychiatry.

[20] Almeida OP, Almeida SA (1999). Short versions of the geriatric depression scale: a study of their validity for the diagnosis of a major depressive episode according to ICD-10 and DSM-IV. Int J Geriatr Psychiatry.

[21] Ramos LR, Toniolo J, Cendoroglo MS, Garcia JT, Najas MS, Perracini M (1998). Two-year follow-up study of elderly residents in S. Paulo, Brazil: methodology and preliminary results. Rev Saude Publica.

[22] Pereira LV, Pereira GD, Moura LA, Fernandes RR (2015). Pain intensity among institutionalized elderly: a comparison between numerical scales and verbal descriptors. Rev Esc Enferm USP.

[23] Saraiva MD, Suzuki GS, Lin SM, de Andrade DC, Jacob-Filho W, Suemoto CK (2018). Persistent pain is a risk factor for frailty: a systematic review and meta-analysis from prospective longitudinal studies. Age Ageing.

[24] Treede R-D, Rief W, Barke A, Aziz Q, Bennett MI, Benoliel R (2019). Chronic pain as a symptom or a disease: the IASP classification of chronic pain for the international classification of diseases (ICD-11). Pain..

[25] da Motta TS, Gambaro RC, Santos FC (2015). Pain measurement in the elderly: evaluation of psychometric properties of the geriatric pain measure _ Portuguese version. Revista Dor.

[26] Ferrell BA, Stein WM, Beck JC (2000). The geriatric pain measure: validity, reliability and factor analysis. J Am Geriatr Soc.

[27] Ensrud KE, Ewing SK, Cawthon PM, Fink HA, Taylor BC, Cauley JA (2009). A comparison of frailty indexes for the prediction of falls, disability, fractures, and mortality in older men. J Am Geriatr Soc.

[28] Morley JE, Malmstrom TK, Miller DK (2012). A simple frailty questionnaire (FRAIL) predicts outcomes in middle aged African Americans. J Nutr Health Aging.

[29] Aprahamian I, de Castro Cezar NO, Izbicki R, Lin SM, Paulo DL, Fattori A (2017). Screening for frailty with the FRAIL scale: A comparison with the phenotype criteria. J Am Med Dir Assoc.

[30] Fried LP, Tangen CM, Walston J, Newman AB, Hirsch C, Gottdiener J (2001). Frailty in older adults: evidence for a phenotype. J Gerontol A Biol Sci Med Sci.

[31] Lin SM, Aliberti MJR, Fortes-Filho S, de Araújo Melo J, Aprahamian I, Suemoto CK (2018). Comparison of 3 Frailty Instruments in a Geriatric Acute Care Setting in a Low-Middle Income Country. J Am Med Dir Assoc.

[32] Malmstrom TK, Miller DK, Morley JE (2014). A comparison of four frailty models. J Am Geriatr Soc.

[33] Kiely DK, Cupples LA, Lipsitz LA (2009). Validation and comparison of two frailty indexes: the MOBILIZE Boston study. J Am Geriatr Soc.

[34] Reis MM, Arantes PMM (2011). Medida da força de preensão manual- validade e confiabilidade do dinamômetro saehan. Fisioterapia e Pesquisa.

[35] Bohannon R (2002). Quantitative testing of muscle strength: issues and practical options for the geriatric population. Top Geriatr Rehabil.

[36] Eggermont LHP, Leveille SG, Shi L, Kiely DK, Shmerling RH, Jones RN (2014). Pain characteristics associated with the onset of disability in older adults: the maintenance of balance, independent living, intellect, and zest in the elderly Boston study. J Am Geriatr Soc.

[37] Pereira SRM, Buksman S, Perracini M, Py L, Barreto KML, Leite VMM. Quedas em idosos: Projeto Diretrizes. Associação Médica Brasileira,Conselho Federal de Medicina, Sociedade Brasileira de Geriatria e Gerontologia. 2001. http://www.projetodiretrizes.org.br/projeto_diretrizes/082.pdf. Accessed 10 May 2020.

[38] von Elm E, Altman DG, Egger M, Pocock SJ, Gøtzsche PC, Vandenbroucke JP (2007). The strengthening the reporting of observational studies in epidemiology (STROBE) statement: guidelines for reporting observational studies. Ann Intern Med.

[39] Andrade JM, Duarte YA, Alves LC, Andrade FCD, Souza JPRB, Lima-Costa MF (2018). Frailty profile in Brazilian older adults: ELSI-Brazil. Rev Saude Publica.

[40] Nunes BP, Batista SRR, Andrade FB, Souza JPRB, Lima-Costa MF, Facchini LA (2018). Multimorbidity: The Brazilian Longitudinal Study of Aging (ELSI-Brazil). Rev Saude Publica.

[41] Giacomin KC, Duarte YAO, Camarano AA, Nunes DP, Fernandes D (2018). Care and functional disabilities in daily activities - ELSI-Brazil. Rev Saude Publica.

[42] Lebrão ML, Duarte YA, JLF S, Silva NN. 10 Anos do Estudo SABE: antecedentes, metodologia e organização do estudo. Rev Bras Epidemiol. 2018;21. 10.1590/1980-549720180002.supl.2 30726347

[43] Duarte YA, Nunes DP, Andrade FB, Corona LP, Brito TRP, Santos JLF, et al. Fragilidade em idosos no município de São Paulo: prevalência e fatores associados. Rev Bras Epidemiol. 2018;21. 10.1590/1980-549720180021.supl.2 30726366

[44] Alves LC, Leimann BCQ, Vasconcelos MEL, Carvalho MS, Vasconcelos AGG, da Fonseca TCO (2007). A influência das doenças crônicas na capacidade funcional dos idosos do Município de São Paulo, Brasil. Cad Saúde Pública.

[45] Lebrão ML, Laurenti R (2005). Saúde, bem-estar e envelhecimento: o estudo SABE no Município de São Paulo. Rev Bras Epidemiol.

[46] Lei X, Sun X, Strauss J, Zhao Y, Yang G, Hu P, et al. Health outcomes and socio-economic status among the mid-aged and elderly in China: evidence from the CHARLS national baseline data. J Econ Ageing 2014;3:29–43. doi:https://doi.org/10.1016/j.jeoa.2014.05.001. 10.1016/j.jeoa.2014.10.001 PMC6699996 31428556

[47] Smith JP, Strauss J, Zhao Y (2014). Healthy aging in China. J Econ Ageing.

[48] Zhao Y, Hu Y, Smith JP, Strauss J, Yang G (2014). Cohort profile: the China health and retirement longitudinal study (CHARLS). Int J Epidemiol.

[49] Wu C, Smit E, Xue Q-L, Odden MC (2017). Prevalence and correlates of frailty among community-dwelling Chinese older adults: the China health and retirement longitudinal study. J Gerontol Ser A.

[50] Garcia-Pena C, Avila-Funes JA, Dent E, Gutierrez-Robledo L, Perez-Zepeda M (2016). Frailty prevalence and associated factors in the Mexican health and aging study: A comparison of the frailty index and the phenotype. Exp Gerontol.

[51] Wong R, Michaels-Obregon A, Palloni A (2017). Cohort profile: the Mexican health and aging study (MHAS). Int J Epidemiol.

