# Supplementary material for: Prospective GERiatric Observational (ProGERO) study: cohort design and preliminary results
Source: BMC Geriatr. 2020 Oct 27;20:427. doi: 10.1186/s12877-020-01820-4 (PMC7590705; doi:10.1186/s12877-020-01820-4)
Supplement: Supplementary file 1 — Additional file 1. Research Protocol – ProGERO Study. [file 12877_2020_1820_MOESM1_ESM.docx]

| PATIENT LABEL |
| --- |

| **Additional File**  **RESEARCH PROTOCOL – ProGERO Study** | | |
| --- | --- | --- |
| **N^O^ __________** | **Date: ______ / ______ / ______** | Informant:🞏 patient 🞏 legal guardian: _________________ |

| **SOCIODEMOGRAPHIC DATA** | | | | | | | | | | | | | | |
| --- | --- | --- | --- | --- | --- | --- | --- | --- | --- | --- | --- | --- | --- | --- |
| Name: | __________________________________________________________ | | | | | | | Hospital ID: | | _____________________ | | | | |
| Sex: 🞏 F 🞏 M | | Date of Birth: ____ / ____ / ____ | | | Age: _____ (years) | | | | Have caregiver? | | | 🞏 Yes 🞏 No | | |
| Race:______________ | | | | | | | | Level of literacy: _______ (years) | | | | | | |
| Main occupation : _______________________________________________ | | | | | | | | Retired:🞏 Yes 🞏 No | | | | | | |
| Civil status: ______________________________ | | | | | | | | Live alone?: 🞏 Yes 🞏 No | | | | | | |
|  | | | | | | | | With whom?___________________ | | | | | | |
| Adress: _______________________________________________________ | | | | | | | | N^o^ _________ | | | Compl. __________ | | | |
| Neighborhood: ____________ | | | City: ___________________ | | | State: | ______ | Zip Code:_____________________ | | | | | | |
| Caregiver name: ____________________________________ 🞏F 🞏M | | | | | | | |  | | | | | | |
| Age: _______ (years) | | | Scholarity: _______ (years) | | | | |  | | | | | | |
| Type (family member, tutor, curator): ________________________________ | | | | | | | | | | | | | | |
| Do the patient has contact with children at home? 🞏 Yes 🞏 No  If Yes, how many children? __________How old are them? ________________ _____ | | | | | | | | | | | | | | |
| **TELEPHONES** | | | | | | | | | | | | | | |
| Residential 1: ____________________________________ | | | | | | | | | | | | |  |  |
| Residential 2: ____________________________________ | | | | | | | | | | | | |  |  |
| Cell phone 1: ____________________________________ | | | | | | | | | | | | |  |  |
| Cell phone 2:____________________________________ | | | | | | | | | | | | |  |  |
| Email: | | | | | | | | | | | | |  |  |
| **Annual household income** | | | | | | | | | | | | | | |
| Family income: _______________ | | | | How many people live at home? _______________ | | | | | | | | | | |

**RECENT USE OF HEALTH SYSTEM**

| 1. Over the past six months, have you been hospitalized for ≥ 24 hours? | 🞏 Yes 🞏 No |
| --- | --- |
| 1. If yes, how many times? __________________________ 2. Did the hospitalization occur due to an acute illness? 🞏 Yes 🞏 No | |
| c. Do you know the hospitalization diagnoses? __________________________________________ | |
| 2. Over the past six months, have you been to emergency room (ER)? | 🞏 Yes 🞏 No |
| a. If yes, how many times? __________________________ | |

| **BOMFAQ – Brazilian OARS Multidimensional Functional Assessment Questionnaire** | | | |
| --- | --- | --- | --- |
| 🞏 patient 🞏 caregiver | | | |
| **For the tasks described below, are you able to realize them:** | **With no help** | **With help** | **Unable to realize** |
| 1. Pour and out of bed | 2 | 1 | 0 |
| 1. Eat | 2 | 1 | 0 |
| 1. Comb the hair | 2 | 1 | 0 |
| 1. Walk in the flat | 2 | 1 | 0 |
| 1. Shower | 2 | 1 | 0 |
| 1. Dress up | 2 | 1 | 0 |
| 1. Going to the bathroom (continence) | 2 | 1 | 0 |
| 1. Cut toenails | 2 | 1 | 0 |
| 1. Taking medicines on time | 2 | 1 | 0 |
| 1. Get around in places close to home | 2 | 1 | 0 |
| 1. Go shopping | 2 | 1 | 0 |
| 1. Prepare meals | 2 | 1 | 0 |
| 1. Climb a flight of steps | 2 | 1 | 0 |
| 1. Take transportation | 2 | 1 | 0 |
| 1. Clean the house | 2 | 1 | 0 |
| **TOTAL =** |  | | |

| 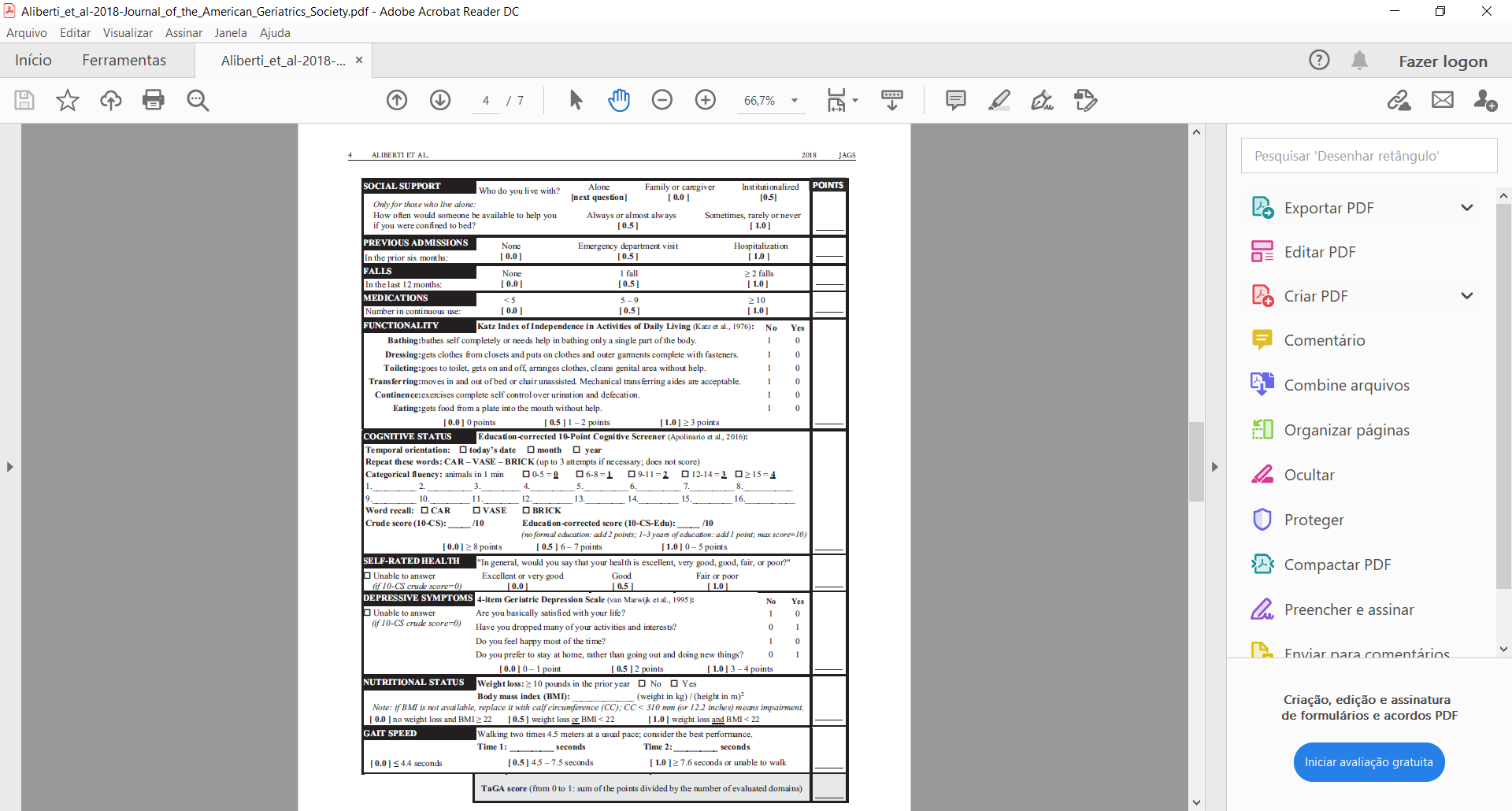 |
| --- |

**COMORBIDITIES**

| 1. Anemia | 🞏 Yes 🞏 No | 14. Chronic liver disease | 🞏 Yes 🞏 No |
| --- | --- | --- | --- |
| 2. Rheumatoid arthritis | 🞏 Yes 🞏 No | 15. Hypertension | 🞏 Yes 🞏 No |
| 3. Dementia | 🞏 Yes 🞏 No | 16. Peripheral Arterial insufficiency | 🞏 Yes 🞏 No |
| 4. Depression | 🞏 Yes 🞏 No | 17. Congestive heart failure | 🞏 Yes 🞏 No |
| 5. Diabetes Mellitus | 🞏 Yes 🞏 No | 18. Coronary disease - Angina | 🞏 Yes 🞏 No |
| 6. Renal Dysfunction | 🞏 Yes 🞏 No | 19. Coronary disease - Infarction | 🞏 Yes 🞏 No |
| 7. Cerebrovascular Disease | 🞏 Yes 🞏 No | 20. Obesity (BMI ≥ 30) | 🞏 Yes 🞏 No |
| 8. Chronic obstructive pulmonary disease | 🞏 Yes 🞏 No | 21. Osteoarthritis | 🞏 Yes 🞏 No |
| 9. Asthma | 🞏 Yes 🞏 No | 22. Osteoporosis | 🞏 Yes 🞏 No |
| 10. Gastroesophageal Reflux Disease | 🞏 Yes 🞏 No | 23. Parkinson or Parkinsonism | 🞏 Yes 🞏 No |
| 11. Aortic stenosis | 🞏 Yes 🞏 No | 24. Generalized Anxiety Disorder | 🞏 Yes 🞏 No |
| 12. Chronic Atrial Fibrillation | 🞏 Yes 🞏 No | 25. HIV/AIDS | 🞏 Yes 🞏 No |
| 13. Smoking 🞏 Yes 🞏 No 🞏 Stopped | ____ years ago | 26. Cancer, wich? | 🞏 Yes 🞏 No |
| Packs a day: Total time: _____ anos |  | Current treatment? | 🞏 Yes 🞏 No |
| NUMBER OF DISEASES = |  | 26. Alcohol abusel? 🞏 Yes 🞏 No Type:______  🞏 Stopped: _____years ago Total time: _____ anos | Dose per day:_____ |

**Other comorbidities: _____________________________________________________________________________________________________________________________________________________________________________________________________________________________________________________________________________________________**

**Medications in use (name and dosage)**

**______________________________________________________________________________________________________________________________________________________________________________________________**

**______________________________________________________________________________________________________________________________________________________________________________________________**

**______________________________________________________________________________________________________________________________________________________________________________________________**

**______________________________________________________________________________________________________________________________________________________________________________________________**

**Physical examination and performance; frailty, sensory and pain evaluation**

PHYSICAL EXAMINATION AND PERFORMANCE:

Blood pressure: 1)________ 2)_______ 3)________

Pulse rate: 1)________ 2)_______ 3)________

Handgrip strength: 1)________ 2)_______ 3)________ 🞏Unable

Chair-stands test: ________ 🞏 Unable

FRAILTY:

1. SOF:

Weight loss: “How much do you weigh with your clothes on but without shoes? [current weight]” “One year ago in (MO, YR), how much did you weigh without your shoes and with your clothes on? [weight 1 year ago]”

Percent weight change is computed as: [[weight 1 year ago - current weight]/weight 1 year ago]] * 100. Percent change > 5 (representing a 5% loss of weight) is scored as 1 and < 5 as 0.

Exhaustion: “Do you feel full of energy?” 1 = No, 0 = Yes

Low mobility: Inability to perform a chair rise five times 1 = Yes, 0 = No

1. FRAIL:

Fatigue: “How much of the time during the past 4 weeks did you feel tired?”

1 = All of the time, 2 = Most of the time, 3 = Some of the time, 4 = A little of the time, 5 = None of the time.

Responses of “1” or “2” are scored as 1 and all others as 0.

Resistance: “By yourself and not using aids, do you have any difficulty walking up 10 steps without resting?”

1 = Yes, 0 = No.

Ambulation: By yourself and not using aids, do you have any difficulty walking several hundred yards?”

1 = Yes, 0 = No.

Illnesses: For 11 illnesses, participants are asked, “Did a doctor ever tell you that you have [illness]?”

1 = Yes, 0 = No. The total illnesses (0–11) are recoded as 0–4 = 0 and 5–11 = 1. The illnesses include hypertension, diabetes, cancer (other than a minor skin cancer), chronic lung disease, heart attack, congestive heart failure, angina, asthma, arthritis, stroke, and kidney disease.

Loss of weight: “How much do you weigh with your clothes on but without shoes? [current weight]” “One year ago in (MO, YR), how much did you weigh without your shoes and with your clothes on? [weight 1 year ago]”

Percent weight change is computed as: [[weight 1 year ago - current weight]/weight 1 year ago]] * 100. Percent change > 5 (representing a 5% loss of weight) is scored as 1 and < 5 as 0.

SENSORY:

Can you see well enough to recognize a friend across the street? 🞏 Yes 🞏 No

Can you usually hear and understand another person when they talk in a normal voice? 🞏 Yes 🞏 No

PAIN:

Did you have any pain in the last 3 months? How intense was the pain (5-point Verbal Descriptor Scale)?

🞏no pain; 🞏mild pain; 🞏moderate pain; 🞏severe pain; or 🞏worst possible pain

Did the pain occur on most days in the past three months? 🞏 Yes 🞏 No

_____________________________________

Researcher Signature
